# Validation of a Bioanalytical Method for the Determination of Synthetic and Natural Cannabinoids (New Psychoactive Substances) in Oral Fluid Samples by Means of HPLC-MS/MS

**DOI:** 10.3389/fchem.2020.00439

**Published:** 2020-06-05

**Authors:** Luca Calò, Luca Anzillotti, Chiara Maccari, Rossana Cecchi, Roberta Andreoli

**Affiliations:** ^1^Legal Medicine, Department of Medicine and Surgery, University of Parma, Parma, Italy; ^2^Laboratory of Industrial Toxicology, Department of Medicine and Surgery, University of Parma, Parma, Italy; ^3^Centre for Research in Toxicology (CERT), University of Parma, Parma, Italy

**Keywords:** new psychoactive substances, biological matrices, liquid chromatography, mass spectrometry, oral fluid, drugs of abuse, bioanalysis, method validation

## Abstract

New psychoactive substances (NPS) represent an important focus nowadays and are continually produced with minimal structural modifications in order to circumvent the law and increase the difficulty of identifying them. Moreover, since there are a high number of different compounds, it is arduous to develop analytical screening and/or confirmation methods that allow the identification and quantification of these compounds. The aim of this work is to develop and validate a bioanalytical method for detecting new synthetic drugs in biological samples, specifically oral fluid, using high-performance liquid chromatography coupled with mass spectrometry (HPLC-MS/MS) with minimal sample pretreatment. Oral fluid samples were simply centrifuged and denaturized with different rapid procedures before injection into the LC-MS/MS system. Calibration curves covered a linear concentration range from LOQ to 100 ng/mL. Validation parameters such as linearity, precision, accuracy, selectivity, matrix effect and thermal stability were evaluated and showed satisfactory results, in accordance with US Food & Drug Administration guidelines. The inter-day analytical bias and imprecision at two levels of quality control (QC) were within ±15% for most compounds. This method was able to identify and calculate the concentration of 10 NPS validated in this biological sample, even in the presence of matrix effect.

## Introduction

The drugs of abuse scenario is constantly changing with the rapid development of unregulated synthetic and ad-hoc-designed compounds (Anzillotti et al., 2019b; UNODC Early Warning Advisory on New Psychoactive Substances, 2020). In recent years, new psychoactive substances (NPS), including synthetic cannabinoids, as well as semi-synthetic opioids and heroin, have emerged as chemical compounds the use of which is spreading very rapidly, not only in the United States of America but also in European countries, in addition to the use of classic drugs. Their popularity derives—especially in young generations—from their low cost and the high number of online shops. NPS are continually synthesized in illegal laboratories, and their consumption produces significant dangerous effects on human health that are still under investigation, such as agitation, aggression, and acute psychosis, as well as the potential development of dependence or cardiovascular effects (Anzillotti et al., 2019a; UNODC Early Warning Advisory on New Psychoactive Substances, 2020). In this context, the development and validation of analytical methods able to rapidly identify and correctly quantify such compounds are highly encouraged from a clinical and scientific point of view.

The purity and composition of products containing NPS are often unknown, and it can be very difficult to develop methods to identify them in biological matrices (Strano-Rossi et al., 2012, 2014; Brunt et al., 2017; Anzillotti et al., 2018, 2019b; Bianchi et al., 2019; Graziano et al., 2019; Williams et al., 2019). Although, at present, there is a rapid development of these substances of abuse, there are no current guidelines with threshold reference standard values for NPS in biological samples, to the best of our knowledge. In several countries, controls on groups of these “legal” substances are carried out using the principle of “chemical similarity” with substances already under the regulatory mechanism (United Nations Office on Drugs Crime, 2019). These substances have become a global phenomenon, with more than 120 countries and territories having reported at least one NPS (UNODC Early Warning Advisory on New Psychoactive Substances, 2020). In particular, until recently, 950 substances have been reported to the (UNODC) by governments, laboratories, and partner organizations (Rocchi et al., 2018; United Nations Office on Drugs Crime, 2019, 2020). Although synthetic cannabinoids are rapidly evolving and there are serious difficulties controlling them on the market (Coulter et al., 2011), in Europe, the European Monitoring Centre for Drugs and Drug Addiction (EMCDDA), through the EU Early Warning System, constantly monitors the use of synthetic cannabinoids. For the first time in 2008, forensic investigators in Germany and Austria took over the synthetic cannabinoid JWH018 (European Monitoring Centre for Drugs Drug Addiction, 2015). Since the 2000s, the synthetic cannabinoids were sold as mixtures for smokers, resins, and mixtures containing other psychoactive substances such as stimulants, hallucinogens, sedatives, and hypnotics. A recent development has been the discovery of synthetic cannabinoids in liquids for electronic cigarettes, now very popular among young people (European Monitoring Centre for Drugs Drug Addiction, 2017).

Therefore, the continuous challenge for forensic toxicologists is the identification of NPS in classical and alternative biological matrices such as oral fluid (Bosker and Huestis, 2009; Anizan and Huestis, 2013; Patsalos and Berry, 2013). The struggle for forensic laboratories is also due to the unknown composition of these new substances: therefore, the treatment of a patient who has taken synthetic drugs, perhaps in combination with other substances or alcohol, is quite difficult (Governo Italiano, 2017; Busardo et al., 2018). To date from a recent search of the literature, only a few studies have dealt with the determination of such compounds in oral fluid; however, thanks to current technological progress in the forensic toxicology field, a few screening tests and more selective techniques for alternative matrices have been developed, though the modern literature on analytical methodologies applied to these matrices is still limited, and a more detailed validation is often required (Gallardo and Queiroz, 2008; Strano-Rossi et al., 2012, 2014; Huestis et al., 2017; Anzillotti et al., 2018, 2019b; Bianchi et al., 2019; Graziano et al., 2019). Oral fluid has gained popularity as an alternative to classical hematic and urinary approaches in the field of workplace drug testing and the testing of drivers for being under the influence of drugs owing to its ease of collection and reduced detection time windows (Strano-Rossi et al., 2012; Edvardsen et al., 2015; Anzillotti et al., 2019b; Bianchi et al., 2019; Graziano et al., 2019). In particular, it appears to be an easier matrix to analyze and allows the rapid identification of recently developed drugs (Mercolini and Protti, 2016). No invasive sampling and easy storage are additional advantages deriving from the use of oral fluid over traditional matrices like blood or urine (Anzillotti et al., 2019b).

The aim of this article is to evaluate the best experimental conditions (in terms of sample pretreatment, technical parameters, and compound stability) in which to use oral fluid as an alternative matrix to detect these emerging drugs of abuse with a newly validated bioanalytical method.

## Experimental

### Chemicals and Reagents

Water, acetonitrile (AcCN), formic acid, and methanol (MeOH) were purchased from Sigma Aldrich (Milan, Italy).

Cannabinol (6,6,9-trimethyl-3h pentyl-6H-dibenzo[b,d] pyran-1-ol), cannabidiol (2-[(1R,6R)-3-methyl-6-(prop-1-en-2-yl) cyclohex-2-en-1-yl]-5-pentylbenzene-1,3-diol), THC (Δ9-tetrahydrocannabinol), THCCOOH (11-Nor-9-carboxy-Δ9-tetrahydrocannabinol), UR144 (1-pentyl-1H-indol- 3-yl) (2,2,3,3-tetramethylcyclopropyl)methanone, CP47497-C7 (2-[(1R,3S)-3-hydroxycyclohexyl]-5-(2-methyl-2-octanyl)phenol) and its homolog CP47497-C8 (2-(3-hydroxycyclohexyl)-5-(2-methylnonan-2-yl)phenol), AM2201 (1-([5-fluoropentyl]-1H-indol-3-yl)-(naphthalen-1-yl)methanone), JWH019 [(1-hexyl-1H-indol-3-yl)-1-naphthalenyl-methanone], JWH081 (4-methoxy-1-naphthalenyl 1-pentyl-1H-indol-3-yl) methanone), JWH122 (4-methyl-1-naphthalenyl) (1-pentyl-1H-indol-3-yl)methanone), JWH250 (1-(1-pentyl-1Hindol-3-yl)-2-(2-methoxyphenyl)-ethanone), JWH200 ([1-(2-morpholin-4-ylethyl)indol-3-yl]-naphthalen-1-ylmethanone), mephedrone or MEPH [(RS)-2-methylamino-1-(4-methylphenyl)propan-1-one], HU211 [(6As,10As)-9-(Hydroxymethyl)-6,6-dimethyl-3-(2-methyloctan-2-yl)-6a,7,10,10a-tetrahydrobenzo[c]chromen-1-ol], and Δ9-tetrahydrocannabinol-D3 (THC-D3) were also supplied from Sigma Aldrich (Milan, Italy). Standard compounds were stored according to supplier recommendations until their use. Amicon Ultra 3K devices (Millipore, Billerica, MA, USA) were used to centrifuge oral fluid samples.

### Sample Collection and Preparation

Oral fluid samples were collected from 25 healthy volunteers free of drugs of abuse (both males and females), after obtaining their informed consent. The oral fluid was collected in a 15 ml plastic tube without an identification number in order to avoid any possible identification of the donor. Each sample was centrifuged at 4,000 rpm for 10 min. We mixed five different supernatants to obtain a pooled oral fluid sample. Five samples set with the minimum volume necessary for all the method validation experiments were prepared. The pooled lots were aliquoted and stored at −20°C until analysis (Gruppo Tossicologi Forensi Italiani, 2017).

All the experiments described below were prepared starting from these five pooled oral fluid samples, and each experiment was performed on all the five pooled lots independently.

Sample preparation consisted of combining 95 μL of pooled oral fluid with 5 μL of ISTD (THC-D3 at a concentration of 10 ug/mL) and 200 μL of MeOH in plastic vials; the samples were centrifuged at 10,000 rpm for 5 min, and, finally, 100 μL of supernatant was transferred to other plastic vials and injected into the HPLC-MS/MS system.

### HPLC-MS/MS Equipment

The HPLC instrument was an Agilent Series 1100 (Agilent Technologies, Santa Clara, CA, USA) equipped with a degasser, a binary high-pressure gradient pump, and a thermostatted autosampler module. The MS/MS instrument was an API4000 (SCIEX, Framingham, MA USA) equipped with a TurboIonSpray interface for pneumatically assisted electrospray. Separation of the analytes and the internal standard (THC-D3) was performed using an Agilent® Pursuit XRs Ultra 2.8 C18 (100 × 2.0 mm) column, with mobile phase A consisting of an aqueous component (10 Mm formic acid in water at pH 3,75) and organic phase B (methanol/acetonitrile, 95/5 V/V) with the addition of 10 Mm formic acid, operating in an elution gradient. Elution gradient: 15% organic phase B, hold for 2 min; from 15 to 80% organic phase B in 1.5 min (linear gradient), hold for 1 min; from 80 to 100% organic phase B in 1 min (linear gradient); 100% organic phase B, hold for 5,5 min; then back to the starting condition in 0.5 min and re-equilibration for 13.5 min. The flow-rate was 0.2 ml min^−1^. The injection volume was 2 μl, and each analysis required 25 min, including the re-equilibration time. The first (0–3 min) and the last (22–25 min) parts of the chromatographic run were diverted to waste using a 10-port valve (Valco Systems, Houston, Texas, USA).

### Method Development and Validation

#### Mass Spectrometer Parameters

A mixture of analytes at a concentration of 0.2 μg/mL was infused in the mass spectrometer (an API 4000 Sciex coupled with an Agilent 1100 LC system, as mentioned above) both for optimizing physical parameters such as declustering potential (DP) and collision energy (CE) and for detecting characteristic analyte transitions. In Q1 mode, the MS parameters, such as gas temperature, gas flow, capillary voltage, and declustering potential, were optimized in order to obtain higher sensitivity for the [M+H]^+^ as precursor ion. In product ion scan mode, the parameters were set to obtain the best signal to noise ratio for the fragments of each studied compound. The analyte ionization was obtained in positive mode and acquired in the Selected Reaction Monitoring (SRM) mode.

In Table 1, the retention times, mass spectrometer parameters, and optimized MRM transitions are summarized. The most abundant transition was chosen as the quantifier ion (Q) and the second and third, if present, most abundant transitions as qualifiers (q1 or q2). The q/Q ratio (<20%) was calculated to provide an additional identification criterion besides the retention time, as per forensic requirements.

**Table 1:** Time window setting [Retention time (RT), Q1, parent ion; declustering potential (DP), Q3, ions resulting from ion parent fragmentation, quantifier ion (Q); qualifier ion (q), collision energy (CE)] for liquid chromatography–tandem mass spectrometry analysis.

**Compound**	**RT**	**[M+H]^**+**^**	**DP**	**Q**		**q1**		**q2**	
				***m/z***	**CE**	***m/z***	**CE**	***m/z***	**CE**
MEPH	9.8	178	50	160	20	145	30	119	30
JWH200	11.9	385	90	155	25	114	35	298	25
AM2201	12.9	360	90	155	35	127	55	144	55
JWH250	13.4	336	90	121	30	91	45	144	20
CP47497-C7	13.6	319	40	233	20	175	15	301	15
CP47497-C8	13.6	333	40	315	15	247	20	175	15
CBD	13.7	315	90	193	30	259	25	-	-
THCCOOH	13.8	345	90	193	30	299	40	327	30
JWH081	14.1	372	90	185	35	214	35	157	35
JWH122	14.3	356	90	169	35	141	35	214	55
JWH019	14.3	356	90	155	35	127	55	228	35
UR144	14.4	312	90	125	35	214	35	144	45
CBN	14.8	311	70	223	30	214	25	195	35
HU221	15.1	387	90	243	25	261	25	-	-
THC	15.3	315	90	193	30	259	30	123	30

*RT, Retention Time; [M+H]^+^, precursor ion; DP, declustering potential; Q, quantifier ion; q1 and q2, qualifier ions; CE, collision energy*.

#### Sample Treatment

Three different denaturation methods were compared in order to characterize the effect of sample treatment on signal intensity: (a) centrifugation of 500 μL of oral fluid with plastic centricon vials (3kDa filter) at 14,000 rpm for 15 min; (b) chemical denaturation of 100 μL of biological sample with an equal volume of AcCN, followed by centrifugation at 10,000 rpm for 5 min; (c) chemical denaturation of 50 μL of oral fluid with 100 μL of methanol, followed by centrifugation at 10000 rpm for 5 min. All three different protein denaturation procedures were applied on five pooled lots by spiking aliquots at the different calibration levels.

A pooled oral fluid sample was prepared by mixing five different oral fluid samples collected from five different healthy subjects and centrifuged at 4,000 rpm for 10 min. Each pooled sample was divided into seven different aliquots, each one spiked with the levels prepared for the calibration curve (L0-L6; see below for details). The samples, with different NPS concentrations, were further divided into three different aliquots in order to obtain three sets of samples, each following a different denaturing treatment.

#### Method Validation

Method validation parameters, such as linearity, sensitivity, accuracy, precision, and thermal and storage stability, were studied following FDA guidelines. In particular, linearity, sensitivity, accuracy, and precision were calculated in water and in the pooled matrices; thermal and storage stability were calculated in matrix as well. Relative matrix effects were examined by using the approach proposed by Matuszewski: slope CV (%), the slope difference (%), and the assay CV range (%) (Matuszewski, 2006; Food Drug Administration (FDA), 2018) were obtained from five different lots of biological fluid spiked with the same calibration levels.

##### Pool oral fluid experiment

About 2 mL of oral fluid was collected from five volunteers, both men and women, and were mixed to make a pool of 10 mL in order to evaluate the influence of the different sources. Subsequently, the pool was used to prepare the same concentration levels. Two different conditions were tested for the calibration curve in water: 50/50 v/v MeOH/HCOOH 0.1 M and 50/50 v/v MeOH/H_2_O. The oral fluid pool levels were prepared in 50/50 MeOH/HCOOH 0.1 M in both plastic and glass vials in order to evaluate whether the vial composition could interfere with analyte detection. After centrifugation (10,000 rpm for 5 min), 2 microliters of all levels were injected into the HPLC-MS/MS.

Two different levels and a blank (0, 5, and 10 ng/mL) were prepared in plastic and glass vials with different conditions: 70/30 MeOH/H_2_O, 50/50 MeOH/H_2_O, and 100% MeOH.

Moreover, five oral fluid pools were prepared by mixing different oral fluid samples collected from different subjects (*n* = 10). Hence, each pool was used to prepare a calibration curve (0, 1, 5, 10, 20, 50, and 100 ng/mL) with three different final conditions: 3 kDa centricon vials, 1:1 acetonitrile, and 1:2 MeOH. This trial was also replicated in water as control.

##### Linearity, sensibility, accuracy, and precision

The linearity of the method was calculated by preparing six calibration levels from the standard material mixture in order to obtain final concentration levels of 0, 1, 5, 10, 20, 50, and 100 ng/mL in water and further verified by homoscedastic test. To evaluate the sensitivity, the limit of detection (LOD) as a signal/noise ratio >3 (S/N>3) and the limit of quantification (LOQ) as a S/N>10 were calculated, preparing further diluted samples in water.

The between- and within-run accuracy and precision of the method based on back-calculated concentration, were evaluated by analyzing 10 solvent blank samples spiked at two different concentration levels – QC 1 (low, 5 ng/mL), QC 2 (high, 50 ng/mL) – over a 5-day validation period. The values of accuracy, CV% intraday (*n* = 4), and CV% inter-day (*n* = 6) were considered acceptable if CV% <15%. All samples prepared in water for the calculation of linearity, sensibility, accuracy, and precision were combined with a double volume of MeOH, as described in the sample preparation paragraph, before injection into the LC-MS/MS system. All of the experiments relating to the method validation parameters were repeated in the five pooled lots.

##### Matrix effect

The absolute matrix effect (ME) was determined as follows: set 1 was composed of five replicates of the calibration curve levels diluted in water; set 2 was composed of five oral fluid blank pooled samples fortified with analytes after protein denaturation at the same concentration as the replicates of set 1, for each analyte and concentration. ME was calculated by dividing the mean peak areas of set 2 by those of set 1 (Matuszewski et al., 2003).

The relative matrix effects were estimated by following the standard procedure proposed by Matuszewski (2006) by calculating the slope CV (%), the slope difference (%), and the assay CV range (%) obtained from five different lots of blank biological fluid pools spiked with the same calibration levels. Slope CV (%) is the precision of standard line slopes constructed in five different batches of a biofluid, expressed as a coefficient of variation. A cut-off value of lower than 3–4% has been suggested to establish whether the method is practically free from a significant relative matrix effect. Slope difference (%) is calculated as

(1) $$\begin{array}{l} \text{Slope difference }\left(\%\right)=\left(\frac{\text{Slop}{\text{e}}_{\text{max}}-\text{Slop}{\text{e}}_{\text{min}}}{\text{Slop}{\text{e}}_{\text{min}}}\right)\% \end{array}$$

and corresponds to the maximum difference in the calculated concentration of an analyte in five batches studied that originates from the relative matrix effect. Finally, “assay CV range (%)” is the range of coefficient of variation values determined at all concentrations used for constructing standard lines. It represents the overall method precision and should not exceed 8.7% in the absence of relative matrix effects (Matuszewski, 2006).

##### Thermal stability

*Freeze/thaw cycles experiment* Analyte stabilities after freeze/thaw cycles (*n* = 5) and after storage were assessed by percentage change from T0, calculated by the formula:

(2) $$\begin{array}{l} \left[\left(C_{\text{X}}-C_{\text{T}0}\right)/C_{\text{T}0}\right)]\times \;100\% \end{array}$$

where C_T0_ is the back-calculated concentration of the T0 sample and C_X_ is the back-calculated concentration of the Tx samples. QC1 and QC2 samples, prepared as described above, were divided into five aliquots (I, II, III, IV, and V) and frozen at −20°C in box A for each pool. After the first cycle, all the aliquots were defrosted, and only aliquot I was transferred into box B; then, both boxes were stored at −20°C. For the second cycle, only box A was defrosted, aliquot II was transferred into box B, and box A was re-frozen, and so on until aliquot V, so that each aliquot went under a different and increased number of freeze/thaw cycles. After that, 100 μL of each sample was added to 200 μL of MeOH, which was centrifugated, and then the supernatant was injected into the HPLC-MS/MS.

*Thermal stability in the short storage condition* Two series of eight aliquots from the QC1 and QC2 levels were prepared and stored at room temperature and at +4°C for 0, 2, 4, 19.3, 23.5, 27.5, 43.5, and 47.5 h, respectively, to test short-term thermal and storage stability, before the longer storage at −20°C. After 1 week, 100 μL of each vial was thawed and combined with 200 μL of MeOH and centrifuged, and the supernatant was injected into the HPLC-MS/MS. In both experiments, thermal stability was tested by comparing the instrument response of the two series with that immediately frozen after the pool preparation (T = 0). For quantitative purpose, a decrease in sensibility of <15% was considered acceptable.

##### Storage condition

In order to evaluate whether the material composition of the vial could interfere with analyte detection, after the denaturation step, we aliquoted the samples prepared for the “matrix effect experiment,” both in water and in the five different pools, in two different types of vial: glass vials and plastic vials, all for laboratory use and LC-MS/MS analyses. The samples were then injected into the HPLC-MS/MS.

## Results and Discussion

### Denaturation Method

Optimization of sample preparation plays a crucial role in the method validation procedure, in particular when the biological matrix is rich in both inorganic and organic compounds (such as proteins) and is quite unknown in terms of quantitative analysis. A denaturation process was carried out to remove the protein component from the matrix samples, with the intent of minimizing the sample treatment as well.

The choice of the denaturation method took into account parameters such as the sensitivity, linearity, affinity of the solvent with ionization efficiency, chemical and physical matrix interactions, and sample density. Three different denaturation procedures were compared with appropriate experiments: (a) ultrafiltration with a cut-off of 3 KDa, based on a physic interaction, (b) dilution with an equal volume of AcCN, as our routine for plasma samples; (c) dilution with two volumes of MeOH, a solvent that normally increases the ionization efficiency.

To study the different behavior of the compounds as a result of the denaturation procedures, we prepared the calibration curves both in water and in five different oral fluid pools. Then, each calibration level was divided into three different aliquots, each one treated with denaturation procedures (a), (b), and (c), respectively. All samples were centrifuged, and the supernatants were injected into the LC-MS/MS system.

We observed that by using the ultrafiltration devices, only MEPH was detectable amongst all other compounds, probably since the physical denaturation was not adequate to break the interactions of the synthetic cannabinoids with the oral fluid components or the stronger affinity of aminoalkylindole compounds for oral fluid proteins with respect to the amphetamine-like compounds.

Chemical denaturation methods, obtained with AcCN or MeOH, were both adequate to detect all the compounds of interest; however, denaturation with AcCN, even if with a lower dilution factor, was less sensitive and exhibited higher analytic variability compared to the procedure with MeOH.

The accuracy of the denaturation procedure was determined as the average of the accuracies, defined as the ratio between the recalculated value and the true value, of each level and for all the compounds. For the denaturation procedure using AcCN, this accounted for 122% for samples obtained in water and 132% for spiked oral fluid ones, which were both higher than the 80–120% range assessed in the FDA guidelines. However, when MeOH was used as the solvent for protein precipitation, the accuracy for water calibrations was 115% and 116% for the same samples but with NPS mixture added to the real matrices. Moreover, the precision of the experiment, calculated as the average of CV% for all calibration levels and for all analytes, was 21.1 and 10.9% for water samples and 22.2% and 17.5% for pooled ones if denaturized with AcCN and MeOH, respectively.

In addition, comparing the response of the compounds diluted in water or spiked in pooled oral fluid, we observed a signal intensity decrease when samples were denaturized with AcCN instead of MeOH. The lower dilution factor, and the consequently higher amount of matrix components, may reduce the efficiency of the ionization process when samples are treated with AcCN instead of MeOH.

### Validation Parameters in Water

Linearity, sensitivity, accuracy, precision, matrix effects, and thermal and storage stability were the criteria assessed for the method validation, following the Guidance for Industry Bioanalytical Method Validation of the FDA (2001). The accuracy and precision values were all within the acceptable limit of < 15%, except for CP47497-C7 and -C8. The linearity of the method, in the range from LOQ to 100 ng/mL, was confirmed for the compounds of interest by the calculation of correlation coefficients (*R*^2^ > 0.991) with six levels for five repeated injections, except for CP47497-C7 and -C8 and JWH250, for which the *R*^2^ was calculated with only four concentration levels.

Calibration curves were constructed by linear regression analysis of the area analyte versus the concentration of analytes injected (no IS) and also by linear regression analysis of the area analyte ratios analyte/IS versus the concentration of analytes injected (with IS). Moreover, Hartley's test, the Cochran test, and minimum variance tests were applied to verify hetero/homoscedasticity; all tests obtained results within limits and were comparable to tabulated parameters, certifying homoscedasticity.

The LODs and LOQs were selected based on the lowest concentration with S/ *n* = 3 and S/ *n* = 10, respectively. For JWH250, CP47497-C7, and CP47497-C8 we observed a higher LOD (> 1 ng/mL), so only QC2 accuracy and precision were calculated (Samano et al., 2014).

The inter-day mean accuracy (%) of the analytes, calculated without the use of IS and at the two spiking levels, was in the range of 85–107%, with the exception of CP47497-C8, which was 146%. The same parameter, calculated with the use of the IS, was reduced to have a range of 89.7–108%, but it increased to 164% for CP47497-C8. The range of the inter-day mean precision (%) of the analytes was 0.85–13.1 and 0.60–12% when the values were calculated without considering or with considering the IS, respectively. Again, for CP47497-C8, the inter-day mean precision (19.1%) exceeded the FDA guidelines criteria of a value lower than 15%, independently from the use of IS. The accuracy and precision values were also calculated in intra-day experiments, ranging between 92–103% and being below 10%, respectively. The method validation criteria such as sensitivity, accuracy, and precision (inter-day) at two concentrations (QC1 5 ng/mL and QC2 50 ng/mL), calculated both without and with IS for each compound are reported in Table 2.

**Table 2:** Method validation criteria, sensitivity, accuracy, and precision (inter-day) at two quality control concentrations (5 and 50 ng/mL), calculated both without and with IS, for each compound added to water.

**Water**	LoD*	LoQ*	**Accuracy%**				**Precision%**			
			**QC1**		**QC2**		**QC1**		**QC2**	
***Compound***	***ng/mL***	***ng/mL***	***No IS***	***With IS***	***No IS***	***With IS***	***No IS***	***With IS***	***No IS***	***With IS***
MEPH	25.4	84.5	90.9	101.0	102.0	106.0	4.4	5.6	4.2	4.3
JWH200	55.7	185.0	91.6	102.0	103.0	106.0	4.0	5.3	2.8	3.0
AM2201	1.8	6.0	85.0	94.5	97.9	101.3	3.1	4.4	3.0	2.5
JWH250	378.0	1259.0	99.5	111.0	103.0	107.0	5.6	5.8	0.9	1.5
CP47497-C7	831.0	2769.0			99.0	103.0			9.8	10.0
CP47497-C8	599.0	1965.0			146.0	162.0			19.0	19.0
CBD	90.1	300.0	85.6	95.3	94.5	97.8	12.6	13.5	5.0	5.5
THCCOOH	190.0	635.0	85.7	96.0	100.0	105.0	2.5	2.8	3.9	4.6
JWH081	0.9	2.9	87.8	97.6	107.0	111.0	13.1	13.4	12.3	12.3
JWH122	1.1	3.6	88.9	98.9	106.0	110.0	4.7	5.4	2.0	2.6
JWH019	1.5	5.0	92.0	102.0	106.0	110.0	4.2	5.7	3.4	3.3
UR144	1.4	4.6	86.4	96.1	100.0	104.0	4.2	5.5	2.3	2.7
CBN	103.0	343.0	87.9	97.8	94.8	98.1	7.2	7.9	6.1	6.6
HU221	76.6	255.0	94.0	105.0	92.9	96.3	2.9	1.3	2.3	2.5
THC	102.0	342.0	86.2	95.9	94.8	98.1	2.4	4.0	2.3	2.8

LOD*, Limit of detection x10^−3^, S/N = 3; LOQ*, Limit of quantification x10^−3^, S/N = 10; QC1, 5 ng/mL; QC2, 2, 50 ng/mL; No IS, results obtained with calibration curves constructed by linear regression analysis of the analyte area vs. the concentration of analytes injected; With IS, results obtained with calibration curves constructed by linear regression analysis of the ratio of the analyte area to the IS area vs. the concentration of analytes injected.

Data are reported both with and without IS, and the IS was chosen due to chemical structure similarity and the commercial availability of the deuterated IS; our aim was also to verify whether this approach could be applicable for routine analysis in order to define method reliability, robustness, and accuracy. In general, without IS, we observed lower accuracy with low concentration samples, as expected (85.0–99.1); on the other hand, method accuracy showed an opposite trend: for most compounds, inter- and intra-day CVs% were lower without IS, although CVs% were lower than 15%, as required by the guidelines, even with IS.

### Validation Parameters in Oral Fluid

The experiments were then replicated in oral fluid; the results are summarized in Table 3. In general, we noticed that for JWH250, CP47497-C7, and CP47497-C8, LODs and LOQs were higher than for the other compounds, similarly to the results in water. Similar results were obtained with IS or without IS, as shown in the table; therefore, Tables 2, 3 show similar results.

**Table 3:** Method validation criteria, sensitivity, accuracy, and precision (inter-day) at two quality control concentrations (5 and 50 ng/mL), calculated both without and with IS, for each compound added to five different oral fluid pools.

**Matrix**	LoD*	LoQ*	**Accuracy%**				**Precision%**			
			**QC1**		**QC2**		**QC1**		**QC2**	
***Compound***	***ng/mL***	***ng/mL***	***No IS***	***With IS***	***No IS***	***With IS***	***No IS***	***With IS***	***No IS***	***With IS***
MEPH	173.0	575.0	97.6	91.9	104.0	103.0	4.2	6.6	3.0	6.2
JWH200	82.4	275.0	103.0	96.2	108.0	107.0	3.0	5.0	1.8	4.0
AM2201	2.8	9.2	101.0	93.4	111.0	108.0	3.5	3.1	3.7	2.7
JWH250	166.0	553.0	118.0	109.0	118.0	115.0	3.3	4.8	4.4	4.5
CP47497-C7	2275.0	7583.0			122.0	118.0			6.5	9.0
CP47497-C8	422.0	1406.0			171.0	161.0			7.1	9.8
CBD	83.6	279.0	97.4	89.4	112.0	109.0	7.0	10.1	4.8	5.9
THCCOOH	116.0	386.0	99.1	92.6	106.0	104.0	9.2	7.7	3.7	5.5
JWH081	1.6	5.5	109.0	99.7	111.0	109.0	3.7	5.5	5.0	6.1
JWH122	1.0	3.3	114.0	104.0	113.0	110.0	2.2	4.6	2.3	4.6
JWH019	5.3	17.7	112.0	104.0	113.0	110.0	3.0	5.6	3.9	4.5
UR144	2.0	6.6	108.0	99.9	110.0	107.0	2.4	5.8	3.3	6.8
CBN	146.0	487.0	102.0	92.0	112.0	108.0	10.6	11.7	8.3	12.0
HU221	173.0	575.0	113.0	105.0	111.0	108.0	6.5	7.3	7.0	5.7
THC	82.4	275.0	102.0	93.8	104.0	100.0	8.1	6.9	7.4	8.4

LOD*, Limit of detection x10^−3^, S/N = 3; LOQ*, Limit of quantification x10^−3^, S/N = 10; QC1, 5 ng/mL; QC2, 50 ng/mL; No IS, results obtained with calibration curves constructed by linear regression analysis of the analyte area vs. the concentration of analytes injected; With IS, results obtained with calibration curves constructed by linear regression analysis of the ratio of the analyte area to the IS area vs. the concentration of analytes injected.

However, particularly in terms of accuracy, the results show more variability without IS for QC1 (89.3 and 105% in water and oral fluid, respectively) and QC2 (103.2 and 115%, respectively), while with IS, these differences tend to decrease. A different outcome is seen for precision since the variability seems to be IS-dependent rather than concentration-dependent. LODs and LOQs showed better results in terms of sensibility in water samples with respect to oral fluid samples, so we were able to calculate QC1 values as well.

### Matrix Effect

Table 4 shows the results obtained from both absolute (confronting water/matrix samples) and relative matrix effect experiments carried out confronting the same matrices but coming from different sources and therefore with different endogenous compounds and/or interferent concentrations.

**Table 4:** Absolute matrix effect (ME%) and relative Matrix effect, obtained on water and on five different oral fluid pools, according to the protocol proposed by Matuszewski (2006).

**Compound**	**ME (%)^a^**		**SLOPE CV%**^b^		**SLOPE DIFFERENCE%**^c^		**ASSAY CV RANGE%**^d^	
	***NO IS***	***With IS***	***NO IS***	***With IS***	***NO IS***	***With IS***	***NO IS***	***With IS***
MEPH	63.8	57.3	19.4	20.3	58.6	58.6	0.40–12.2	1.06–11.9
JWH200	121.0	109.0	13.5	21.6	38.9	69.0	0.77–15.7	0.72–11.2
AM2201	123.0	111.0	13.1	14.2	38.7	48.0	0.38–13.1	0.72–8.30
JWH250	122.0	109.0	11.4	14.1	34.6	44.3	0.35–14.2	0.33–8.56
CP47497-C7	138.0	124.0	20.5	10.2	55.6	30.5	1.81–21.2	0.42–19.1
CP47497-C8	130.0	117.0	18.8	8.7	56.5	26.4	2.15–53.1	2.36–53.3
CBD	114.0	102.0	20.6	12.8	57.7	42.7	0.12–15.3	2.54–16.6
THCCOOH	120.0	108.0	17.3	24.6	47.3	77.2	0.97–20.0	1.50-21.1
JWH081	115.0	103.0	18.1	9.8	53.0	30.3	0.38–8.30	1.59–12.7
JWH122	116.0	104.0	13.7	6.9	40.6	19.0	0.48–10.3	1.03–10.9
JWH019	113.0	101.0	15.0	8.4	38.5	25.6	0.57–11.6	0.53–15.8
UR144	115.0	103.0	15.3	9.4	42.1	27.8	0.41–13.3	0.70–16.1
CBN	123.0	111.0	18.5	4.3	59.2	11.1	0.77–19.7	1.17–26.4
HU221	129.0	117.0	14.9	10.7	37.9	34.9	1.13–12.4	2.04–18.7
THC	118.0	106.0	16.6	9.9	45.4	30.5	0.82–14.1	0.47–15.4

a *ME%, absolute matrix effect*,

b *Precision value (coefficient of variation, CV%) of slopes of standard lines constructed in five different oral fluid pools*;

c *maximum difference between the highest and the lowest slope values divided by the lowest slope value and multiplied by 100*;

d *range of coefficient of variation values (method precision) determined on at all concentrations used for constructing standard lines; No IS, results obtained with calibration curves constructed by linear regression analysis of the analyte area vs. the concentration of analytes injected; With IS, results obtained with calibration curves constructed by linear regression analysis of the ratio of the analyte area to the IS area vs. the concentration of analytes injected*.

For the absolute matrix effect (ME%), mephedrone (or MEPH) shows different chemical behavior from all of the other compounds (similar to what was shown above), highlighting a signal suppression with respect to experiments carried out in water. However, for all other compounds, these differences were minimized with the use of IS.

For the relative matrix effect, the parameters hereby calculated clearly underline the error made when interpreting results on real samples while using a calibration curve built on the same matrix but different biological individuals. The greater values, the higher is the chance of miscalculation with the standard external addition method for a quantitative result (calibration in oral fluid samples).

Therefore, as mentioned by Matuszewski (2006) and based on data presented in this manuscript, it is proposed that the precision (CV) value of standard line slopes constructed in five different lots of a biofluid should not exceed 3–4% for the method to be considered practically free from relative matrix effect liability. In addition to high precision values of standard line slopes (<3.4%) constructed in five different lots of a biofluid, the precision values at all concentrations used for the preparation of standard curves and determined in five different lots of a biofluid did not exceed 8.7%. We observed strong relative matrix effects for different compounds,

In particular, for the parameter “Slope difference %” with respect to the “Assay CV%” range, which represented the range of coefficient of variation values determined at all concentrations used for constructing the standard lines, it suggested that the method is quite precise for quantitative aims.

Since no satisfactory results were achieved for JWH250, CP47497-C7, and -C8 (not shown in the following graphs), we focused on the results from the other compounds. As reported by other authors (Schlittenbauer et al., 2015; Ghosh, 2019) it is recommended to perform the “relative” matrix effect experiment during bio-analytical method validation, particularly when a complex biological matrix, such as oral fluid, is used for a quantitative purpose.

### Thermal Stability

#### Freeze/Thaw Cycles

Further experiments were carried out to evaluate the influence of the freeze/thaw cycles and were therefore performed on QC1 and QC2 samples for all of the five oral fluid pools (the results are reported as mean values).

**Figure 1:**
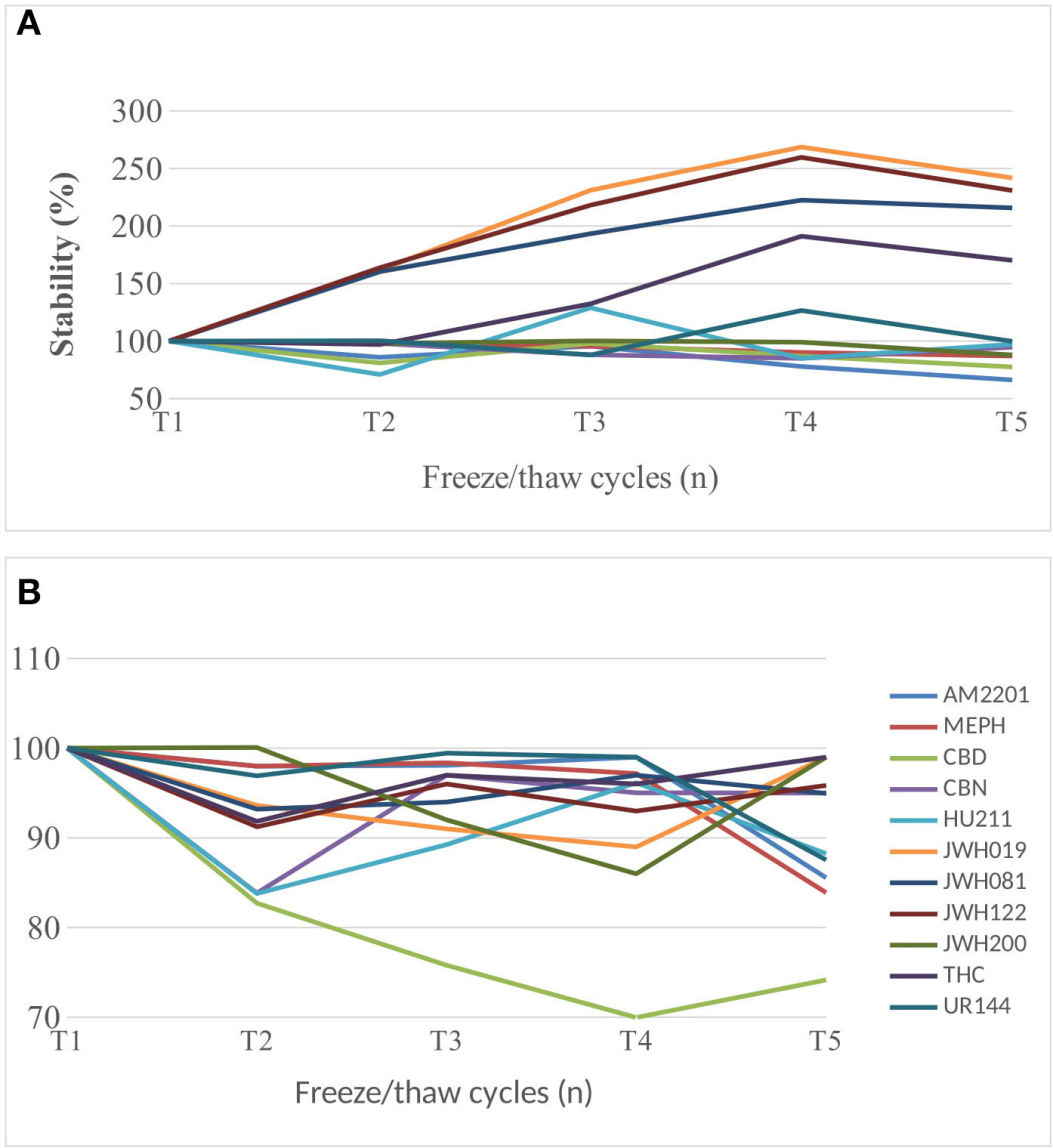
**(A)** QC1 (5 ng/mL) samples in freeze/thaw cycle experiment from T1 to T5 (*n* = 5). **(B)** QC2 (50 ng/mL) samples in freeze/thaw cycle experiment from T1 to T5 (*n* = 5).

At QC1 level, for JWH019, JWH081, JWH122, and THC, we observed a signal increase related to the number of freeze/thaw cycles; while, for the other compounds a small reduction, mainly <20%, was calculated from T1 to T5 (Figure 1A). At higher concentration levels, QC2, all of the analytes showed the same trend, with a progressive decrease in stability (Figure 1B). CBD was the only compound for which the concentration seemed not to be affected by the freeze/thaw cycles; in fact, in both cases, the decrease in stability was equal to 26%.

In these experiments, it was noticed an increase in response related to the number of freeze/thaw cycles (the more, the higher response), especially for low concentration levels, where matrix effects are supposed to have a major impact.

#### Thermal Stability in the Short-Term Storage Condition

**Figure 2:**
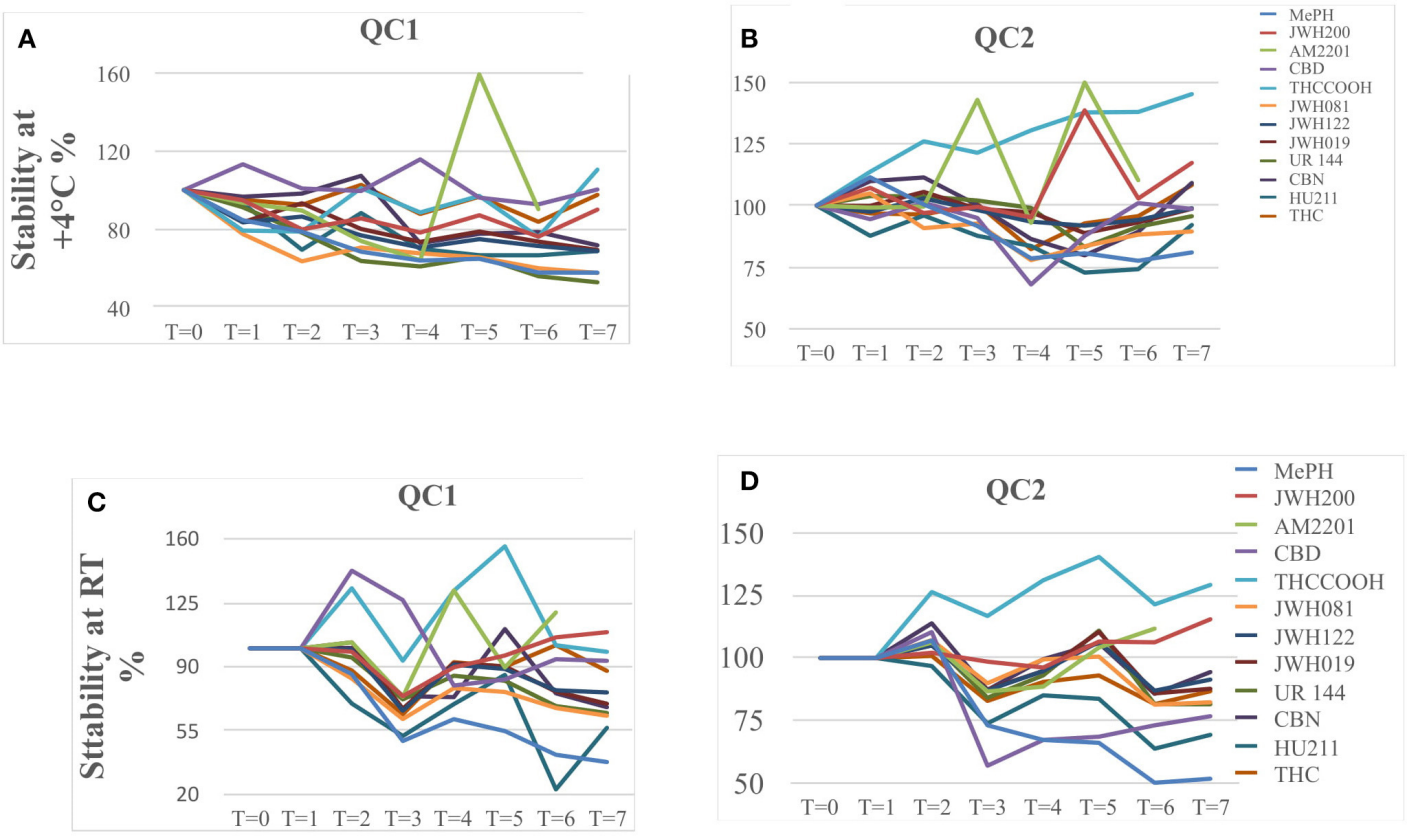
QC1 and QC2 sample stability when stored at +4°C **(A,B)** and at room temperature **(C,D)**.

To obtain data on short-term thermal stability, two series of eight aliquots from QC1 and QC2 levels were added to the five oral fluid pools and were stored one at room temperature and one at +4°C for eight time intervals, 0, 2, 4, 19.3, 23.5, 27.5, 43.5, and 47.5 h, respectively, before the longer-term storage at −20°C, as shown in Figure 2.

Before the experiment, all samples were thawed, and 100 μL of each was combined with 200 μL of MeOH and centrifuged, and the supernatant was injected into the HPLC-MS/MS.

In Figures 2A,B, we report the stability trend of the QC1 and QC2 samples stored at +4°C, respectively, while in Figures 2C,D the trends of the same samples but stored at room temperature are shown. In general, QC2 samples were more stable than QC1 ones, and the same applies for those stored at +4°C as for the samples kept at room temperature.

Already after 24 h, QC1 samples stored at room temperature showed a decrease of <15%, while only JWH200, JWH122, JWH019, and THC registered a decrease of circa 15%, but after 48 h, JWH200 and THC resulted to be stable (<15%).

Concerning the same QC1 samples but stored at +4°C, CBD, THCCOOH, and THC showed a lower decrease in terms of signal intensity of 15%, and therefore, their behavior was considered stable.

In the QC2 samples series, different trends were noticed: in particular, MEPH, CBD, and THCCOOH already showed degradation after 24 h of storage, both at room temperature and at +4°C. When considering 48 h of storage, at room temperature JWH122, JWH019, CBN, and THC were stable, while JWH122, JWH019, UR144, CBN, and THC were stable at +4°C. Data Sheet 1 with raw data can be found in the Supplementary Material.

### Storage Condition

**Figure 3:**
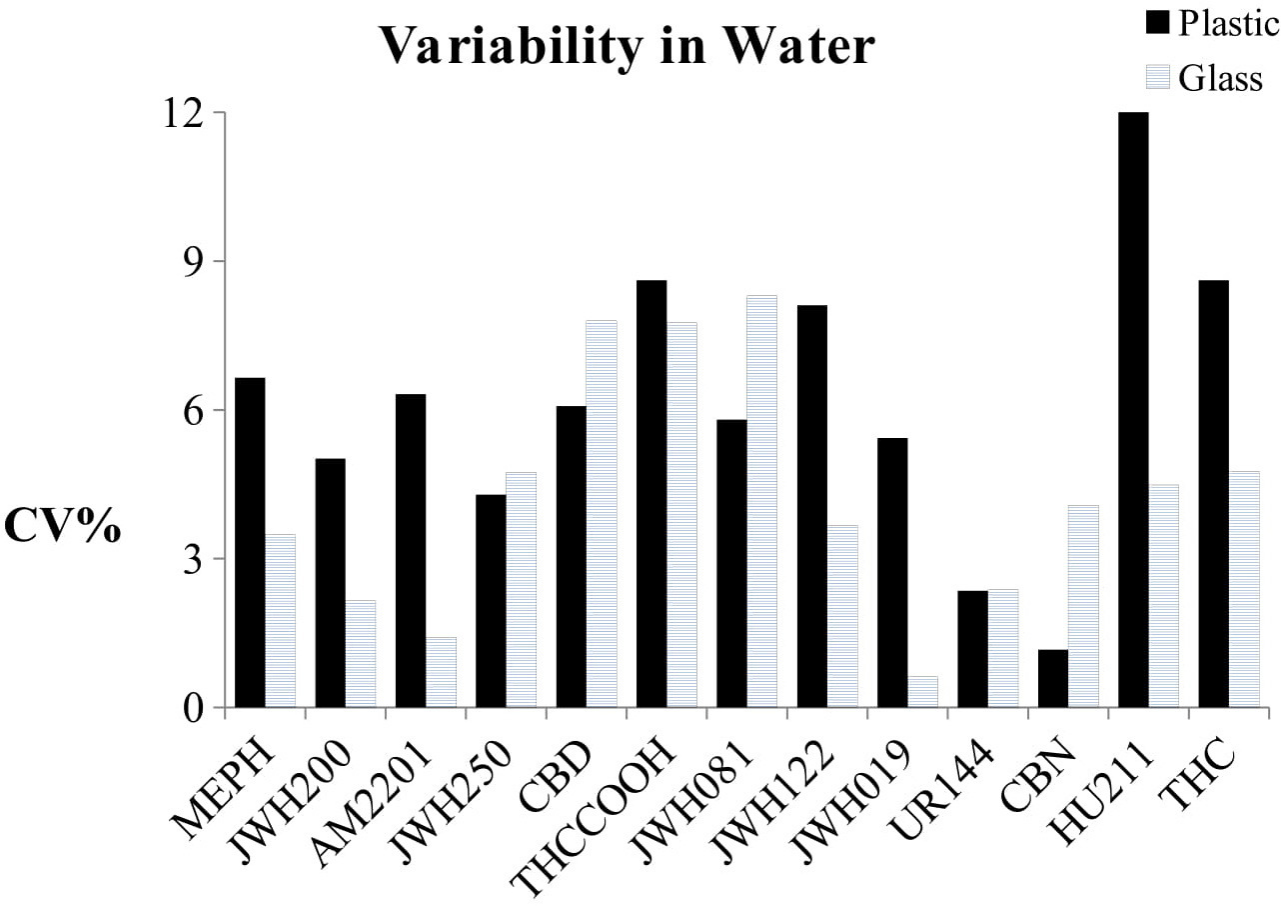
Variability trend in water of samples stored in plastic and glass vials.

**Figure 4:**
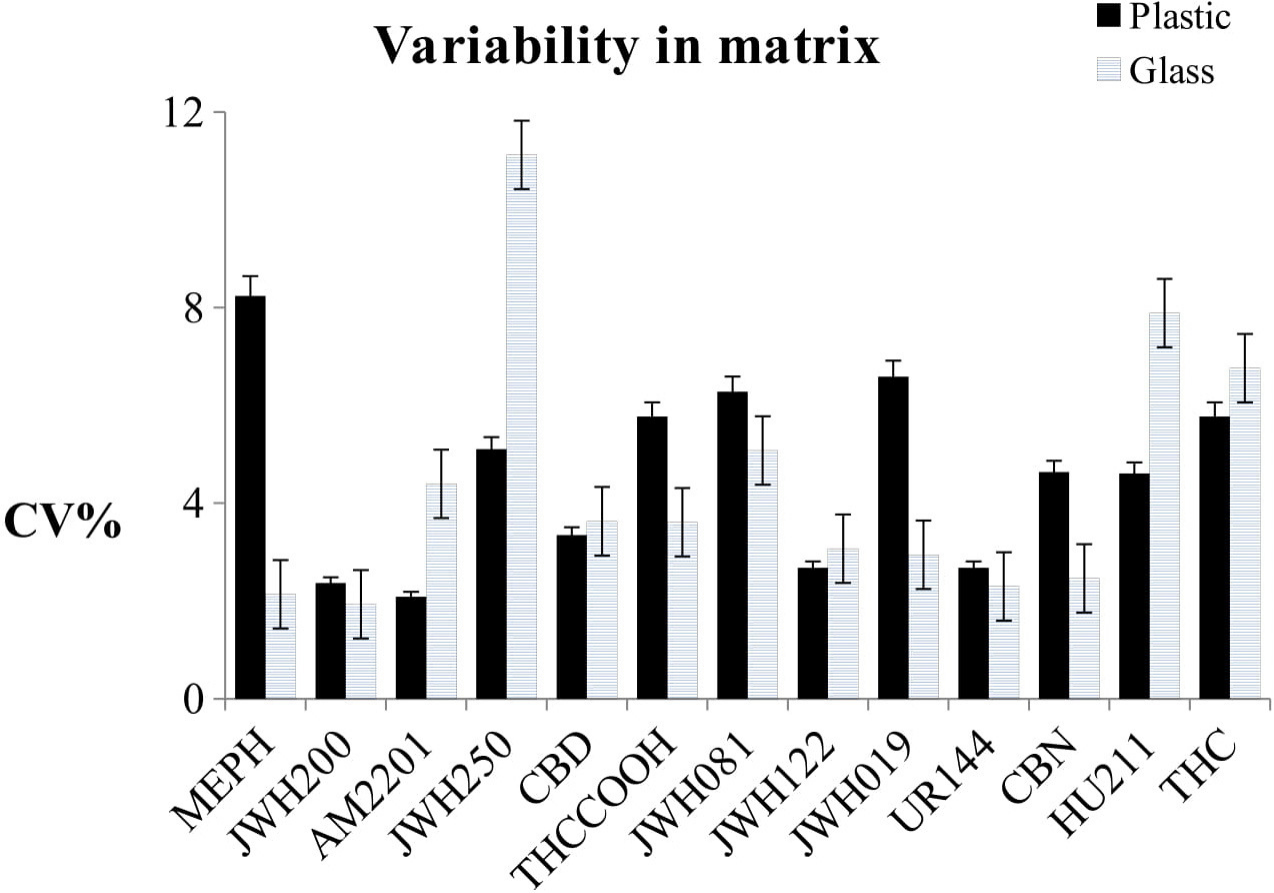
Variability trend in oral fluid samples stored in plastic and glass vials, with uncertainty bands.

The effect of sample storage during the analytical session was evaluated by using the same samples prepared for the matrix experiment (Michelot et al., 2017). After the denaturation and centrifugation steps, all of the sets were divided (one calibration curve prepared in water and five in different oral fluid pools) into two lots: one in plastic vials and the other one in glass (Figures 3, 4). All of the samples were injected in triplicate. Figure 3 shows the variability trend in water of samples stored in plastic and glass vials.

In Figure 4, the uncertainty bands show average results from the five different pools. The variability of the storage condition was calculated as CV% of the slopes of the calibration curves for the samples prepared in water and as the mean of the CV% of the slopes of the calibration curves for the samples prepared in matrix. When the NPS mixture was spiked in water, only CBD and CBN showed higher variability if put in glass vials instead of plastic (CV_glass_/CV_Plastic_%> 15%), and the CVs% relating to JWH250, THCOOH, and UR144 were < 15% and seemed to be not dependent on the material type of the vials.

## Conclusions

Although a significative relative matrix effect was demonstrated during our validation process, the method was fully validated and therefore will be applied to real samples for the determination of 10 NPS in oral fluid with minimal sample pretreatment, reducing matrix effects with the use of the appropriate IS or evaluating the matrix protein content beforehand. However, the method is sensitive, simple, and rapid, and the sample preparation is easy. Moreover, analyte stability at room temperature or lower seems to be concentration-dependent and less dependent on freeze/thaw cycles (with the exception of CBD).

While using glass vials in the validation process, a reduction in measurement variability was noted, and, therefore, the use of glass is encouraged in order to enhance accuracy and precision (apart from for JWH250 and HU211).

The method was validated following guidelines (Food Drug Administration (FDA), 2018) and was demonstrated to be applicable due to its reliability and satisfactory results in a forensic context.

## Data Availability Statement

All datasets generated for this study are included in the article/Supplementary Material.

## Ethics Statement

The studies involving human participants were reviewed and approved by Prot. 39722 01/10/2019 Comitato Etico Area Vasta Emilia Nord. The patients/participants provided their written informed consent to participate in this study.

## Author Contributions

LC: analytical experiments, data analysis and method validation. LA: data review, editing and reviewing of the manuscript, corresponding author. CM: graphics and data analysis. RC: reviewer of analytical experiments and manuscript. RA: reviewer of the manuscript, coordinator of the group, data reviewer and data interpretation.

## Conflict of Interest

The authors declare that the research was conducted in the absence of any commercial or financial relationships that could be construed as a potential conflict of interest.
